# Supplementary material for: Joint regression modeling of blood pressure and associated factors among adults in Uganda: Implications for clinical practice
Source: PLOS Glob Public Health. 2024 Sep 12;4(9):e0003707. doi: 10.1371/journal.pgph.0003707 (PMC11392332; doi:10.1371/journal.pgph.0003707)
Supplement: Attachment — Submitted filename: Response to Reviewers.docx [file pgph.0003707.s001.docx]

**Point-by-Point Response to Reviewer Comments**

**Reviewer #1:**

The paper aims to contribute to the field of epidemiology by examining the sociodemographic and lifestyle factors associated with systolic and diastolic blood pressure among adults in Uganda using a multivariate analysis approach. The use of a large, nationally representative sample from the 2014 Uganda STEPS NCD Risk Factors Survey and the employment of a multi-stage cluster sampling design provide a solid empirical basis for the study. The paper’s originality is evidenced in its focus on a Ugandan population, which adds to the body of knowledge regarding blood pressure variability in a lesser-studied African context. Furthermore, the application of a multivariate linear regression model to identify correlates of systolic and diastolic blood pressure simultaneously is a methodological strength that could yield insights into the multifactorial nature of hypertension.

The paper’s methodology section is a particular strength, as it outlines a robust statistical approach to data analysis including the use of primary sampling unit, strata, and weight adjustments for the complex survey design. Another strength is the relevance of the study to public health, given the growing burden of non-communicable diseases in low- and middle-income countries. The thorough investigation into a wide range of independent variables, including behavioral and metabolic factors, and their relationship with blood pressure, is commendable.

**Response. We thank you for accepting to review our manuscript for the positive feedback/comments.**

**Reviewer #2: Overall impression**

This is a technically well conducted study, but it is unclear what it adds to the scientific literature and does not seem to hold much significance. The authors state multivariate regressions are better/more efficient than univariate, but their own results do not bear this out. In addition, a more robust approach would have been to use simulations to show the difference in precision of estimates between univariate vs multivariate across multiple "datasets", rather than just applying it once to one dataset.

**MAJOR ISSUES**

1. **Introduction**
    Line 58: what kind of biased results? Need more detail here. “Conversely, using multiple linear regression based on the average of one of the blood pressure measurements or a combination of the two blood pressures as independent measurements ignores the association or correlation between SBP and DBP leading to biased results [12].”

**Response: We thank you for this comment. Evidence shows that several independent univariate regression models when used for correlated outcomes such as systolic blood pressure and diastolic blood pressure, the models do not incorporate the association or correlation between the outcomes in the analysis hence leading to biases.**

**In the revised manuscript, we have added the text: “For example, leaving out a correlated outcome leads to omitted variable bias and failure to detect cross-effects between outcomes, overall biasing the regression coefficient estimates. Additionally, the model runs a high risk of producing spuriously significant results as separate Ordinary Least Squares (OLS) regression models neither produce multivariate results nor allow for testing of coefficients across the two equations.”**

2. Line 69: but what is the benefit of “use of more information”? What additional information is gained from simultaneously modelling raised SBP and DBP, than in univariate analysis?

**Response: We appreciate this comment. The additional information or more information in multivariate linear regression analysis includes covariance matrix, cross-effects (effect of one outcome on another outcome), and correlation matrix. Since in multivariate regression analysis, the main focus is to incorporate the correlation between the outcomes, the covariance or correlation matrix as opposed to univariate modeling displays the estimated correlation coefficient between outcomes, highlighting the strength and direction of their relationship potentially leading to a better understanding of the relationship**.

The text has been revised to read as follows: **"Our approach incorporates the association between the two blood pressures thus allowing the use of more information than the commonly used univariate analysis in order to better understand the strength and direction of the relationship."**

3. How is this more efficient? Need to quantify this statement

**Response: Thank you for this comment. As you know, one of the characteristics of a multivariate linear regression model is that the coefficient estimates are generally similar to those from the separate OLS regressions except for the quantification of the residual correlation between SBP and DBP and the testing of the effect of a given risk factor across SBP and DBP. Such is not possible under the separate model results. To demonstrate the efficiency of our approach, we have updated Table 2 with standard errors for both the independent univariate models of SBP and DBP and the joint multivariate response models of SBP and DBP.**

**In the discussion, we have added a new text and it reads: “Our study underscores this efficiency of the analytic approach as reflected by the consistently lower values of standard errors in the joint multivariate response models for SBP and DBP compared to the separate univariate regression models for SBP and DBP.”**

**Methods**

4. Line 115: It’s not clear how the two separate variables SBP and DBP were combined together into one outcome, as described in the Abstract. As it reads, it sounds like the authors kept the SBP and DBP as separate outcome variables

**Response: Thank you for this comment. For descriptive purposes, and to estimate the prevalence of hypertension, a value of 1 was assigned to average SBP ≥ 140 mmHg or average DBP ≥ 90 mmHg (hypertensive), and 0 was assigned to those with average SBP < 140 mmHg or average DBP < 90 mmHg (normotensive). However, as correctly observed, SBP and DBP were kept as separate outcome variables for inferential analysis. To ensure clarity, we have revised the analysis section of the manuscript to explain how SBP and DBP were combined into one outcome variable in order to estimate the prevalence of hypertension. The revised sentence reads as follows: “****To estimate the prevalence of hypertension, a value of 1 was assigned to average SBP ≥ 140 mmHg or average DBP ≥ 90 mmHg (hypertensive) and 0 was assigned to average SBP < 140 mmHg or average DBP < 90 mmHg (normotensive).”**

5. Line 133: why were people with hypertension excluded? Only those on hypertension medications should be excluded

**Response: Thank you for this comment and we agree with your observation. We have revised this part to read as “We excluded people on anti-hypertensive medication”.**

6. Line 141: how was multicollinearity checked? Include details

**Response: Thank you for this comment. We have revised this part in the statistical analysis section to read** **as “To minimize the effect of multicollinearity, only predictor variables with variance inflation factor (VIF) less than 10 were included in the multivariable multivariate model. This was achieved by using the “*Collin”* command (a community-contributed package), a pre-estimation command that displays several different measures of multicollinearity including VIF, and only predictor (independent) variables are used.”**

7. Line 144: there are major problems with variable selection using this method. <https://journalofbigdata.springeropen.com/articles/10.1186/s40537-018-0143-6>. I'd focus on the multivariable regression model (without selecting covariates stepwise, but examining collinearity) and the size of estimated associations, with p-values being secondary.

**Response. Again, thank you. Our understanding is that the model building including the variable selection algorithm for the multivariable regression model including significance testing-based methodology especially with a low alpha level of 5% might omit important confounders. The resources to support this claim are here “Mickey RM, Greenland S. The impact of confounder selection criteria on effect estimation. American journal of epidemiology. 1989 Jan 1;129(1):125-37. Maldonado G, Greenland S. Simulation study of confounder-selection strategies. American journal of epidemiology. 1993 Dec 1;138(11):923-36”. To strike a balance between inclusivity and model parsimony, we set the cut-off p-value of 0.20. However, setting a p-value threshold of 0.2 for predictor selection in exploratory data analysis is a common and acceptable practice as it balances the need for model simplicity and interpretability with the need to avoid overfitting.**

8. Line 175: this section is dense and difficult to follow as a clinical researcher (ie not a statistician). It will be hard to approach for a general audience like PLOS Global Health. What does the matrix mean? What commands did you use in Stata to achieve this. What are the implications of using a matrix, how does it account for the correlation between the response variables?

**Response: We appreciate this comment. In formal multivariate analysis, matrix algebra plays an important role since it greatly simplifies many mathematical expressions. Specifically, Matrices are used to represent covariances and correlations among others. Matrices are essential in multivariate models because they allow compact representation of models and efficient computation (matrix operations are performed quickly enabling faster estimation of parameters). Moreover, matrices facilitate the analysis of covariance and correlation structure between variables. In this study, we solely used a matrix to represent the multivariate model (equation). In Stata, the *mvreg* command with option corr allows estimation of the residual correlation matrix**

**Results**

9. Line 189-191: you said in Methods you excluded people with hypertension. So why would you be presenting the percent of people with hypertension here? It should be 0 if you excluded them.

**Response: Thank you for this comment. We excluded people who are known to be hypertensive and these are individuals on anti-hypertensive medications. The sentence has been revised for clarity and it reads: “We excluded people on anti-hypertensive medication”.**

10. Line 203-212: none of these statistical tests to assess assumptions were explained in Methods. Everything detailed in Results should be mentioned in Methods

**Response: We agree with your observation that statistical tests to assess assumptions were not explained in the methods section. We have revised this section to explain the statistical tests in the methods. The new text reads: “Regression diagnostics were performed to assess the goodness of fit of the multivariate model to data. We assessed the joint significance of the two equations using the F-test to determine the overall effect of the predictor variables on the two responses. The null hypothesis was that the predictor variables overall were not significantly associated with the two responses at a 5% significance level. A resulting p<0.05 led to the rejection of the null hypothesis.** **To test the bivariate normality assumption, the Doornik-Hansen test was employed. Here, we first performed the multivariate regression to obtain the residuals for each outcome and used the *mvtest* normality command with the bivariate option to asses for the bivariate normality of the residuals. The null hypothesis was that the residuals are bivariate normally distributed.** **Lastly, we tested for the independence of the residual correlation using the Breusch-Pagan test. The null hypothesis was that the residuals were not correlated.** **We present the results of these tests in the results section.”**

11. There is nothing here to show/justify why a multivariate model is more efficient/precise than univariate model. Need to compare the results from a multivariate vs univariate model, and show exactly how the multivariate model is “better”. In fact, authors state “Overall, the results are similar to that of the univariate regression model”, so haven’t they proven that the multivariate model is not in fact better?

**Response: The issue of model efficiency has been addressed already. Please refer to the response to comment #3 above.**

**Discussion**

12. Organization here is lacking. First paragraph should focus on this study’s results. Comparison with other studies should be separate paragraphs.

**Response: We are grateful for this guidance. We have revised the discussion section, with the first paragraph focused on the study results and the second paragraph about the comparison of the findings with previous studies.**

13. This focus is all on the risk factors for hypertension, but this is not the point of the study as outlined in the Introduction. The point is that multivariate analysis is better/more efficient than univariate for correlated dependent variables. Thus the discussion should focus on the latter point, and bring in prior statistical work on multivariate vs univariate analyses

**Response: Thank you for this comment. We have revised the discussion section and have included prior statistical work on multivariate and univariate analyses.**

14. Line 287: “The implication of such a positive correlation between SBP and DBP is that either interventions designed to reduce the prevalence of hypertension based on SBP will also reduce the prevalence of hypertension based on DBP.” This is obvious and already known in routine clinical practice. I don’t think this study added much to that understanding.

**Response: Thank you for this comment. We agree with you that interventions to reduce the prevalence of one of the correlated outcomes and also reduce the prevalence of the other in routine clinical practice are already understood. The statement “The implication of such a positive correlation between SBP and DBP is that interventions designed to reduce the prevalence of hypertension based on SBP will also reduce the prevalence of hypertension based on DBP of yet-to-be-identified risk factors” has been deleted from the manuscript**.

15. Line 290: “Furthermore, compared to the naïve separate univariate models, our approach helps control the family-wise error rate - the probability of rejecting at least one of the null hypotheses for a given covariate by testing the effect of a given risk factor across both outcomes simultaneously.” As a non-statistician, I could not understand this sentence. What does “control the family-wise error rate” mean, does multivariate analysis reduce this error rate, or eliminate it? Can you give an example to make this concrete?

**Response. We have revised the sentence using plain language. The revised sentence reads as follows: “Furthermore, the multivariate/joint response modeling approach reduces the occurrence of type I error (the probability of rejecting the null hypothesis that is true in the population) and therefore produces estimates that are highly accurate and reliable compared to modeling such outcomes as separate entities, especially when they are correlated.”**

16. Line 293: “Disregarding this model estimation aspect, as in the fitting of separate regression models for each blood pressure, could lead to imprecision in parameter estimates hencing biasing the results.” How would it bias results? Higher/lower? Also, your results ddn’t show this. Your multivariate models had almost the exact same 95% CI as the univariate, so they were not more precise.

**Response: Thank you for this comment. We have deleted the previous sentence following a revision as shown in response to your comment in #15 just above. We have also already explained the issue of statistical efficiency in response to comment #3 above.**

**MINOR ISSUES**

**Abstract**

17. Used cross-sectional data, cannot use terms like “led to decreased SBP”. Decrease in SBP implies SBP changing in same person over time. Should use “associated with lower SBP”. Same for “increased DBP”
    Introduction

**Response: We agree with your observation. We have revised the abstract and the entire manuscript to use the word “associated” instead of “decreased” or “increased”.**

**Methods**

18. Lines 110-113: this is redundant with text above. Would cut

**Response: Thank you you. This concern was addressed and the new statement under dependent variable now reads: “The current study considered systolic blood pressure and diastolic blood pressure as the two continuous outcome variables measured in units of millimeters of mercury (mmHg).”**

19. Line 116: typo, “and the and these”

**Response: Thank you. This typo was corrected, and any others**

20. Lines 125-131: need to include details of how these variables defined. For example, for physical activity, levels of fasting blood glucose, BMI, etc

**Response: Thank you for the comment. We have revised and included the details of the mentioned variables including hypercholesterolemia as follows:**

**“Physical activity was measured on a binary scale (yes or no) based on whether or not an individual met the WHO recommendations on physical activity for health. Accordingly, adults who had at least 150 minutes of moderate-intensity physical activity, 75 minutes of vigorous-intensity physical activity, or an equivalent combination of moderate and vigorous-intensity physical activity achieving at least 600 milliliters per kilogram per minute were considered physically active. Fasting blood glucose was initially measured as a continuous variable but later categorized as <6.1mmol/l, 6.1-6.9 mmol/l, and ≥7 mmol/l to depict normal fasting blood glucose, impaired, and diabetic status, respectively. Body mass index (BMI) was initially computed as a continuous variable by dividing the weight in kilograms (kg) by the height in meters of an individual, expressed as kilograms per squared meters (kg/m^2^), and later categorized as <18.5kg/m^2^, 18.5-24.9 kg/m^2^, 25.0-29.9 kg/m^2^, and ≥30 kg/m^2^ to denote underweight, normal weight, overweight, and obese respectively. Total blood cholesterol was initially measured as a continuous variable and then categorized as <6.2mmol/L, and ≥6.2mmol/L to depict that the individual had low, normal, and high cholesterol levels, respectively.**

21. Line 134: typo “individual”

**Response: The typo has been corrected.**

**Results**

22. Table 1: hypertension is defined clinically as either SBP >=140 or DBP >= 90. Rather than have separate rows for SBP Hypertensive, and DBP Hypertensive, I’d suggest re-organizing this so it’s Hypertension (yes/no), and then under hypertension have SBP >= 140, DBP >=80, or both as three separate rows

**Response: Thank you for the guidance. Presenting the results in Table 1 for hypertension as suggested would require a cross-tabulation of hypertension (yes vs. no) with SBP ≥140 mm/Hg (yes vs. no) and then DBP ≥90 mm/Hg (yes vs. no). This would make the presentation of the entire results complicated instead of simple descriptive results. Considering this comment, we have revised the presentation of the findings on hypertension for clarity in Table 1. We have provided a clear description, with hypertension stated on a binary scale (yes vs. no) as well as SBP, DBP, and SBP or DBP.**

23. Line 219: Used cross-sectional data, cannot use terms like “increased SBP” as this implies repeated measurements of same individual over time

**Response: We have already addressed this comment. Please see the response to comment # 17.**
