# Supplementary material for: Joint regression modeling of blood pressure and associated factors among adults in Uganda: Implications for clinical practice
Source: PLOS Glob Public Health. 2024 Sep 12;4(9):e0003707. doi: 10.1371/journal.pgph.0003707 (PMC11392332; doi:10.1371/journal.pgph.0003707)
Supplement: Attachment — Submitted filename: Response to reviewers.docx [file pgph.0003707.s002.docx]

**Point-by-Point Response to Reviewer Comments**

**Dear Editor-in-Chief,**

**We thank you and the reviewers for the valuable comments which have helped to improve the quality of the manuscript. We have addressed all the review comments. All changes in the manuscript are tracked. In responding to the review comments, the changes in the manuscript have been populated/replicated and underlined to ease the review of the manuscript. We look forward to receiving a favorable response.**

**Reviewer #3:**

The paper examined determinants of SBP and DBP in Uganda using multivariate multiple regression, accounting for the potential correlation between the two dependent variables. This is the first time I have reviewed the paper and I have focused mainly on the current version of the paper.

**Major comments:**

1. The motivation for using the specific regression technic is sound and implementation appropriate. However, the results were presented mainly as a comparison with separate OLS regressions which is a trivial point because the coefficients should be identical. Instead, the authors should look into more hypothesis-driven research questions, for example to test the difference in the effect of certain determinants on SBP versus DBP, which wouldn't have been possible by using separate OLS regressions.

**Response: We thank you for this comment. In the revised manuscript, particularly in the background, we have added a new text to show that the study is hypothesis-driven. We have revised the main objective to read as follows: :**

**“Therefore,** **we simultaneously model raised SBP and DBP as bivariate and normally distributed data in assessing their risk factors among adults in Uganda. Additionally, we tested the differences in the effect of certain determinants on SBP versus DBP, which would have been impossible under separate OLS regression models”. In the results section, we show findings in Table 3.**

2. I suggest the authors to consider including waist circumference or waist-to-height ratio as an independent variable.

**Response: We thank you for this comment. We have revised the methods section to incorporate the waist-to-hip ratio (we presumed the waist-to-height ratio in the comment is an error) and included it in the analysis and results****. The revised part reads “Central obesity was measured on a binary scale (obese or normal) based on the waist-hip-ratio (waist circumference/hip circumference) of >0.85 in women and >0.95 in men to depict central obesity else normal. During the survey, the waist and hip circumferences were measured to the nearest 0.5 cm using a non-stretchable standard tape measure. The waist measurement was taken mid-way between the lowest rib and the iliac crest with the subject standing at the end of gentle expiration, and hip measurement at the greater trochanters”.**

**We present the findings about waist-hip ratio in Table 1 as well as in Table 2.**

3. Given that DBP tends to decrease in older ages, the author should test the linearity of age effect and potentially include age as either a categorical variable or include a non-linear function of age.

**Response: We appreciate this comment. We tested the linearity effect of age as suggested. The results are shown in Table 3 in the revised manuscript.**

4. The authors should explain how interactions between independent variables were considered.

**Response. We thank you for this comment. We did not focus on the interactions between the independent variables as this was not the main objective of the study. We have included a need for testing interactions between independent variables as a recommendation for future studies. The new text reads:**

**“Interactions between and among the covariates included in this study should be investigated in future studies”.**

5. Given that in theory separate vs joint modelling should give the same results, including SE, I would suggest toning down the mention of the joint modelling showed higher precision or smaller uncertainty throughout abstract, results and discussion. Indeed, the SEs between the two models are only marginally different.

**Response: We agree with your observation that separate vs joint modelling give the same results. We have revised the abstract, results, and discussion sections and toned down the mention of precision or smaller uncertainty.**

**Minor comments:**

6. References #1 and #2 do not support the sentence they were cited for.
   **Response: We are grateful for this observation. We have revised references #1 and #2, and replaced them with correct references that directly support the sentences and they included the following respective references. 1) Alinaitwe B, Amanya C, Muwanguzi PA, Ngabirano TD. Prevalence of Risk Factors for Hypertension Among Faculty at an Urban University in Uganda. Integr Blood Press Control. 2024;17(January 2024):19–20; and, 2) Omar SM, Musa IR, Osman OE, Adam I. Prevalence and associated factors of hypertension among adults in Gadarif in eastern Sudan: A community-based study. BMC Public Health. 2020;20(1):4–9.**
7. The link provided for data access does not provide a direct access to data. Suggest using this instead: [https://extranet.who.int/ncdsmicrodata/index.php/catalog/633/](https://extranet.who.int/ncdsmicrodata/index.php/catalog/633/).
   **Response: Thank you for this comment. We have included the suggested link to the data.**
8. Page 6 Line 133: please explain what "600 milliliters per kilogram" is referring to

**Response: Thank you for this comment. We meant 600 Metabolic Equivalent of Task (MET)-minutes. We have corrected the typo. We have revised the sentence to read as follows:**

**"Accordingly, adults who had at least 150 minutes of moderate-intensity physical activity, 75 minutes of vigorous-intensity physical activity, or an equivalent combination of moderate and vigorous-intensity physical activity achieving at least 600 Metabolic Equivalent of Task (MET)-minutes were considered physically active."**

**We have explained the MET-minutes as follows:**

**“MET-minutes is a unit of measurement used to quantify physical activity. It expresses the total amount of physical activity performed over time (energy expenditure of a specific activity), such as a day or a week. One MET is equivalent to the energy expended at rest and is estimated to be about 1 kilocalorie (kcal)/kg/hour.”**

9. Page 7 Line 141: please correct the sentence: were there two or three categories?
   **Response: Thank you for the guidance. We have revised this part and corrected this typo. The text now reads:**

**“Total blood cholesterol was initially measured as a continuous variable and then categorized as <6.2mmol/L and ≥6.2mmol/L to depict normal (non-hypercholesterolemic) and high cholesterol levels (hypercholesterolemic)”, respectively.**

10. Page 7 Line 151: "or" should be "and"

**Response: We have addressed this comment and replaced “or” with “and”.**

11. Page 8 Line 186: how was complex survey design accounted for, eg through what command or package?

**Response: Thank you for this comment. We employed a combination of Stata and R for our data analysis. To account for the complex survey design and weights, we utilized the 'survey' package in R, which provides a comprehensive set of tools for survey data analysis. Specifically, we used the 'svyglm' function to fit generalized linear models that incorporate the survey weights and design features**. **While Stata was used for initial data cleaning and exploration, R's 'survey' package offered more flexibility and specialized functionality for handling complex survey data.**

12. Page 9 equations: please simplify it to m = 2

**Response: We appreciate this comment. We have simplified the model description to m=2.**

13. Table 2: SE of effect on SBP for impaired FPG in joint model: the number 2.98 is likely a typo - it is the only number that is more than marginally different between the two models.

**Response: The typo has been corrected in the updated/revised Table 2 results.**

14. Table 2: I suggest using normal weight as the reference category for BMI.

**Response: We have re-analyzed the data and used normal weight as the reference category for BMI.**
